# Investigation of the *ZNF804A* gene polymorphism with genetic risk for bipolar disorder in attention deficit hyperactivity disorder

**DOI:** 10.1186/1756-0500-6-29

**Published:** 2013-01-26

**Authors:** Xiaohui Xu, Gerome Breen, Lucy Luo, Bo Sun, Chih-Ken Chen, Ursula M Paredes, Yu-Shu Huang, Yu-Yu Wu, Philip Asherson

**Affiliations:** 1 MRC Social, Genetic and Developmental Psychiatry Centre, Institute of Psychiatry, King’s College London, London, UK; 2 School of Medicine, King’s College London, London, UK; 3 Department of Psychiatry, Chang Gung Memorial Hospital, Taoyuan, Taiwan; 4 Chang Gung University School of Medicine, Taoyuan, Taiwan; 5 Division of Mental Health & Drug Abuse Research, National Health Research Institutes, Taoyuan, Taiwan

**Keywords:** Attention deficit hyperactivity disorder, ZNF804A, Single nucleotide polymorphism, Association study, Bipolar disorder

## Abstract

**Background:**

Genome-wide association studies (GWAS) have been conducted on many psychiatric disorders. Evidence from large GWAS indicates that the single nucleotide polymorphism (SNP) rs1344706 in the zinc-finger protein 804A gene (*ZNF804A*) is associated with psychotic disorders including bipolar disorder and schizophrenia. One study also found significant association between rs1344706 and the executive control network of attention. In this study we examine the role of the rs1344706 polymorphism that previously showed association with BD and is known to alter expression of the gene in two clinical family-based ADHD samples from the UK and Taiwan.

**Findings:**

To investigate the association between rs1344706 and ADHD, two family samples of ADHD probands from the United Kingdom (n = 180) and Taiwan (n = 212) were genotyped using TaqMan SNP genotyping assays and analysed using within-family transmission disequilibrium test. No significant associations were found between rs1344706 polymorphism and ADHD in either of the samples from Taiwan (*P =* 0.91) and UK (*P =* 0.41). Even combining the two datasets together the A allele of rs1344706 SNP was still not significantly over-transmitted to affected probands (*P =* 0.50). Furthermore, there was no evidence of association with the specific symptoms subgroups of inattention or hyperactivity-impulsivity.

**Conclusions:**

In this study we used family-based ADHD data in the UK and Taiwanese population to test for an association between rs1344706 SNP in the *ZNF804A* gene and ADHD. Results showed no significant association of rs1344706 with ADHD in UK and Taiwanese samples.

## Findings

### Background

Attention deficit hyperactivity disorder (ADHD) is one of the most frequent and heritable neurodevelopmental disorders of childhood characterised by developmentally inappropriate and impairing levels of inattention, hyperactivity and impulsivity. Current estimates show that 3%–5% of school age children have a diagnosis of ADHD [1]. The disorder persists into adulthood in around two-thirds of cases [2]. ADHD has been associated with a number of psychiatric comorbidities, including major depressive disorder (MDD), bipolar disorder (BD) and anxiety disorder [3-5]. In particular ADHD and BD present with some overlapping clinical characteristics and diagnostic criteria. Several studies have reported the occurrence of high rates of emotional lability and increased rates of depression in both child and adults patients with ADHD [6-10]. The evidence relating to BD and its potential link with ADHD has been reviewed extensively [5,6] and points to a significant link with evidence from clinical, epidemiological, family and neuroimaging studies. Review of clinical and epidemiological studies found that the increased rate of comorbidity between ADHD and bipolar disorder may be greater than that seen with other psychiatric comorbidities of ADHD [6]. Findings from studies of children with ADHD suggested that combined subtype of ADHD may be at particularly higher risk of developing BD [6,11].

A wide variety of candidate genes have been investigated in ADHD and other psychiatric disorders including BD, with some genes reported to be associated with both ADHD and BD [12-16]. For example, a recent findings support the hypothesis that variation within circadian clock genes contribute to BD and related illnesses like ADHD, MDD and schizophrenia [17]. With regard to ADHD and schizophrenia, there are now several papers showing that rare copy number variants affecting some genes are associated with both ADHD and schizophrenia (SZ) [18-20]. The biological mechanisms that underlie these shared genetic influences remain uncertain, but could be related to common neurodevelopmental processes, or aberrations of neurotransmitter systems such as dopamine.

GWAS studies on ADHD have yet to identify SNPs associations with genome-wide significance [21]. However, promising findings from GWAS have emerged for other psychiatric disorders, particularly SZ and BD. GWAS identified strong evidence for association of the zinc finger protein 804A (*ZNF804A*) gene (located on the long arm of chromosome 2 at the 2q32.1) with psychiatric disorders including both BD and SZ [22,23]. One recent GWAS reported association between rs1344706 in *ZNF804A* and schizophrenia (*P* = 1.61 × 10^-7^), and found strong evidence when the affected phenotype included patients with bipolar disorder (*P* = 9.96 × 10^-9^) [22]. Several replication studies confirmed the association [24-27]. Meta–analysis of a larger dataset found very strong evidence for association between rs1344706 and schizophrenia (*P* = 2.54 × 10^-11^) and a combined schizophrenia/bipolar phenotype (*P* = 4.1 × 10^-13^) [23]. Balog et al., [28] investigated the relationship between rs1344706 in *ZNF804A* and attention in 200 healthy volunteers and found a significant association with a measure of the executive control attention network. One recent study examined six SNPs in five genes in adult ADHD, and concluded that the six SNPs including rs1344706 in *ZNF804A*, which showed strong evidence of association with bipolar disorder, did not appear to be associated with ADHD [29].

As ADHD and BD are both highly heritable, show comorbidity with each other and an increased cross-disorder familial risk [30,31], the aim of the present study was to provide further investigation of genetic evidence linking ADHD and bipolar disorder. We examine the role of the rs1344706 polymorphism in *ZNF804A* in two clinical ADHD samples from the UK and Taiwan.

## Materials and methods

### Subjects

#### The UK ADHD sample

DNA was collected from 180 ADHD combined subtype probands. Both parents were available for 116 families, and only the mother was available for 64 families. Ninety-six percent of the sample was male. Cases were recruited from child behaviour clinics and referred for assessment if they were thought by experienced clinicians to have a diagnosis of combined subtype ADHD under DSM-IV criteria, with no significant Axis I co-morbidity apart from oppositional defiant disorder (ODD, 5.5% of sample) and conduct disorder (CD, 15.5% of sample). Intelligence quotient (IQ) was assessed using the Wechsler Intelligence Scale for Children-Third Edition (WISC-III). Exclusion criteria included IQ less than 70, neurological disorders, brain damage or autism. Only those individuals fulfilling the recruitment criteria after completion of research assessments were included in the study. All probands were of European-Caucasian origin. The age range was 5–15 years at the time of assessment (mean 10.41, SD 2.34). Parents were interviewed with a modified version of the Child and Adolescent Psychiatric Assessment (CAPA) [32]. Information on ADHD symptoms at school was obtained using the teacher Conner’s revised questionnaire [33]. The subjects gave their written informed consent and were approved by the ethical committee of King’s College London (Reference number: G9814668).

#### Taiwanese ADHD sample

Samples were collected from 212 children with ADHD diagnosed between the ages of 5–15 years (mean 8.96, SD 2.60), from both parents for 114 families of the ADHD probands and from only the mother for 59 families or only the father for 39 families. ADHD cases were ascertained from Child Psychiatric Clinics in the Chang Gung Memorial Hospital in Taipei area, Taiwan. The diagnosis of ADHD was made under DSM-IV criteria following completion of a standard maternal interview [34] and completion of Conners’ parent and teacher revised rating scales [33]. Seventy-eight percent of the sample had combined subtype ADHD and 22% had inattentive subtype ADHD, with no co-morbid disorders apart from ODD (5.2%), CD (9.4%) and Tourettes syndrome (1.8%). Cases with autism were excluded from the study. Eighty-nine percent of the sample was male, 87% had IQ greater than 69 and 13% had IQ between 50–69. All subjects gave their written informed consent, and were approved by the Institutional Review Board, Chang Gung Memorial Hospital, Taiwan (Reference number: 96-0058B).

### Genotyping

The rs1344706 polymorphism was genotyped using a TaqMan® SNP genotyping assay (Assay ID: C_2834835_10) on a 7900HT sequence detection system (Applied Biosystems). The distribution of the genotypes did not deviate from Hardy-Weinberg equilibrium (*P* >0.05).

### Statistics

Genotype data from probands and parents were analysed using the transmission disequilibrium test (TDT) implemented in the UNPHASED program (TDTPHASE) [35]. http://www.mrc-bsu.cam.ac.uk/personal/frank/software/unphased/.

## Results

Population frequencies for SNP rs1344706 (an A/C polymorphism) were estimated from parental genotypes. For the UK and the Taiwanese sample the A-allele frequency was 61% and 55%, respectively. There was no significant difference in allele frequencies between the two populations (*χ*^2^ = 0.390, df =1, *P* = 0.474); and genotype frequencies did not deviate from Hardy–Weinberg equilibrium in either population.

TDT analysis (Table 1) showed no evidence of increased transmission of the A allele in either of the samples (Taiwan: *χ*^2^ = 0.012, *P =* 0.91; UK: *χ*^2^ = 0.667, *P =* 0.41). When the two datasets were combined, the A allele was still not significantly over-transmitted to affected probands (*χ*^2^ = 0.458, *P =* 0.50).

**Table 1:** Results of TDT analysis for rs1344706 polymorphism

	**UK samples**		**Taiwanese samples**		**Combined samples**	
	**Allele**		**Allele**		**Allele**	
	**A**	**C**	**A**	**C**	**A**	**C**
Transmitted	44	52	40	41	84	93
Non-transmitted	52	44	41	40	93	84
*χ*^2^, df (*P*-value)	0.667, 1df (0.41)		0.012, 1df (0.91)		0.458, 1df (0.50)	

We further explored the subtypes of ADHD. We found that there was no association between rs1344706 and ADHD in the subgroup of the Taiwanese sample with the inattentive subtype (*χ*^2^ =0.604, *P =* 0.437). We were not able to investigate the hyperactive-impulsive subtype separately because neither samples included any such cases. To investigate whether there were any associations with the specific symptom domains of ADHD, we therefore completed an additional analysis using quantitative ratings of parent rated inattentive and hyperactive-impulsive symptoms using quantitative pedigree disequilibrium test (QPDT) from the UNPHASED program. The results from QPDT analysis showed that the A allele of rs1344706 was not associated with either symptom domain in samples from UK (*Ζ*-score = −0.214; *P =* 0.830 for inattentive symptoms; *Ζ*-score = 0.416; *P =* 0.677 for hyperactive-impulsive symptoms) or Taiwan (*Ζ-*score = 0.253; *P =* 0.801 for inattentive symptoms; *Ζ*-score = 0.220; *P =* 0.826 for hyperactive-impulsive symptoms). When the two datasets were combined, the A allele was still not significantly associated with either symptom domain (*Ζ*-score = −0.293; *P =* 0.769 for inattentive symptoms; *Ζ*-score = 0.049; *P =* 0.961 for hyperactive-impulsive symptoms).

## Discussion

In this study we set out to investigate association between SNP rs1344706 in the *ZNF804A* gene, which was previously found to be associated with BD, and ADHD in two independent ADHD samples from the UK and Taiwan. No association was observed between this polymorphism and ADHD in either sample or when the two samples were pooled together. We further investigated the association on specific symptom subgroups of ADHD. We found no association with the subgroup of the Taiwanese samples with DSM-IV inattentive subtype. Furthermore, using a quantitative trait approach there was no evidence of association with quantitative ratings of either inattentive or hyperactive-impulsive symptoms in either sample.

The human *ZNF804A* gene is located on the long arm of chromosome 2 at the 2q32.1 and *ZNF804A* encodes the transcription factor zinc-finger protein 804A. SNP rs1344706 lies in intron 2 of *ZNF804A* and the A allele is predicted to enhance the maintenance of binding sites for brain-expressed transcription factors [24]. *ZNF804A* gene shows strong evidence of association with both BD and SZ from GWAS [22,23]. The association between SNPs in the Zinc finger gene and ADHD has been suggested before, but a study investigating the relationship between six SNPs reported to be associated with BD, including SNP rs1344706, were not associated in a samples of adults with ADHD. However, a weak association was however observed in the study between rs1344706 and a positive MDQ score, suggesting a possible association with risk for the development of mood symptoms in adult patients with ADHD [29]. Data from another study suggest that rs1344706 is associated with increased response latency on a task assessing the executive control network of attention in healthy adult volunteers [28].

In our study we used family-based ADHD data from the UK and Taiwan to investigate the role between rs1344706 and ADHD. The study presents several limitations that need to be considered. The sample was relatively small and might therefore be underpowered to exclude very small genetic effects. Furthermore, the subgroup with inattentive subtype ADHD was only 22% of the Taiwanese sample and none of the UK sample. Only one polymorphism was genotyped in this gene, therefore we cannot exclude the possibility of association between multiple SNP haplotypes and ADHD.

## Conclusion

This study found no evidence of an association between SNP rs1344706 in *ZNF804A* and ADHD in UK and Taiwanese samples. Due to the limitation of sample size in our study and the fact that, to our knowledge, there is only one published study examining association between rs1344706 and ADHD in adult samples, further association studies are required to investigate the role of the high-risk *ZNF804A* SNPs in ADHD. *ZNF804A* is a protein currently of unknown function. However, the risk allele of rs1344706 associated with other major mental illnesses has been shown to be associated with higher *ZNF804A* expression [24]. Further work should focus on identifying any additional functional polymorphisms of this gene for investigation in further samples of clinical disorders that share genetic risks with either bipolar disorder or schizophrenia.

## Competing interests

The authors declare that they have no competing interests.

## Authors’ contributions

XX selected the SNP, performed genotyping and drafted the manuscript. GB supervised the study and revised the manuscript. LL and BS analysed genetic data. UMP helped to do genotyping. CKC, YSH and YYW provided the Taiwanese DNA samples and clinical data. CKC also revised the manuscript. PA supervised the study and revised the paper. All authors contributed to the final critical revision of the manuscript.
